# The Life of a Travelling Physician, from His First Introduction to Practice, Including Twenty Years' Wanderings through the Greater Part of Europe

**Published:** 1843-07

**Authors:** 


Art. I.—*The Life of a Travelling Physician, from his first introduction to Practice, including Twenty years' Wanderings through the greater part of Europe.* In Three Volumes.—*London,* 1843. 8vo, pp. 312, 304, 294.

This book is not medical; but we notice it on account of its title and of its author, a distinguished member of the profession. Although there are scattered here and there throughout the volumes, brief remarks respecting medicine and medical men, Sir George Lefevre seems to have studiously avoided everything professional in the composition of his book, no doubt intending to favour his own brethren by and bye with an analogous treat to that which he has here given to the general reader. The book is really a very clever one, full of amusing and interesting matter, and giving unequivocal evidence of the author's talent for observation both of men and manners, as well as of a happy facility of graphically delineating what he saw. Although the perusal of these volumes will not add to the medical knowledge of our readers, we can assure them that it will minister not a little to their amusement. As a slight specimen of the author's lively style and manner, we transcribe a few passages from the beginning of the book. Sir George, just dubbed, and fresh from Edinburgh, was delivering his credentials in London, in all the buoyancy of youthful expectation—

"From Pall Mall East I descended a little in the aristocratic scale, and made my next assault upon a dapper little doctor who lived in Bloomsbury Square, to whom I had very strong recommendatory letters. He was the very antipodes of my Scotch friend; he wore powder and silk stockings, and though not very far advanced in his professional course, was, what is styled in medical parlance, a "rising character." He was under the special protection of an old practitioner, who was putting him by degrees into his shoes, which became daily more easy to their new wearer. He was still upon his legs, and had not even launched his *voiture expectante,* yet the profession looked upon him as a rising man. He was established on the neutral ground, or half way between the city and the west-end; and there is a very sensible medical line of demarcation. All is city from Bedford Row eastward. The neutral ground lies between Bedford Row, which it includes, together with the squares to the right, as far down as Charing Cross. All the rest is west. North and south were not marked in the medical chart at that time. Now the neutral ground is very thick set with doctors. It is aspiring ground, and the public judge much of a man's talents by the way in which he seems to thrive himself. The city is decidedly plebeian, the neutral ground aspiring, the west-end aristocratic.

"When young physicians commence in the city, they take a lodging in Fenchurch street, where they generally reside two years upon their private means, if they have the means of residing there so long. They take no fee during this period, but talk very much of their practice increasing, as soon as they have taken one, which is about the beginning of the third year. They talk of having doubled their practice the fourth year, which means that they have taken two fees, and they change their lodgings and remove to Bucklersbury. Here they remain stationary for some time, and if they do not succeed, put their diplomas into their pockets and go into the country to practise as apothecaries. If they succeed, however, and get enough to pay their washerwoman, they take part of a house in Broad street, from whence they remove to the neutral ground and become rising men." (pp. 14-16.)

"I received a letter one morning from my West Indian acquaintance, inviting me to join him at the Italian Opera. I did so, for I was glad of the opportunity; I had never been there before. I was most grievously disappointed. It was between the acts of Il flauto Magico that the doctor turned round to me, and asked me if I would undertake his practice for a fortnight. He wished to go to Sevenoaks, where many of his patients resided, and if I would attend his dispensary, and visit the few patients who remained in town, he would gladly leave them under my care.

"In a few days I was installed in the Doctor's chair, and was myself become a doctor *de facto*. It required more tact to manage the dispensary pupils than the dispensary patients. I found some of these said pupils my seniors in more than age, and very inquisitive. A good face upon difficulties, and carry all with a high hand: I was an advocate for decided practice, as it is styled—a decided practitioner; and there is no more certain way of imposing upon people, than by impressing upon them this idea—say that a man is a decided practitioner, it is enough. Nobody will inquire in what sense—*bad* or good—this word 'decided' is to be taken. I bled, purged, and blistered decidedly, and the cases being of an inflammatory character, as upon Gil Blas' debut, it happened to be decidedly *good* practice.

"I never shall forget the joy which I felt when I fingered the first guinea. It was a genuine coin, for it was at this time, and a most memorable period it was, that I took my maiden fee. The old *unreformed guinea*, none of your sovereigns wrapped up with a shilling, as you see them now-a-days. It was pure and without alloy, and often did I finger it over in my pocket, and sighing involuntary, said to myself, How many more shall I receive in the career which is now opening to me? A conscientious hectic flushed across my face; it was the first and last time that I ever felt embarrassed at receiving a fee. I was in a few days afterwards presented with a second one; it came quite as a thing of course. I thought it tardy in its arrival. These are the only two fees which I took at that period in London." (pp. 19-21.)

